# Genetic variations in the hotspot region of RS1 gene in Indian patients with juvenile X-linked retinoschisis

**Published:** 2007-04-19

**Authors:** Balasubbu Suganthalakshmi, Dhananjay Shukla, Anand Rajendran, Ramasamy Kim, Jeyabalan Nallathambi, Periasamy Sundaresan

**Affiliations:** 1 Department of Genetics, Aravind Medical Research Foundation, Aravind Eye Hospital, Madurai, India; 2 Retina Clinic, Aravind Eye Hospital, Madurai, India

## Abstract

**Purpose:**

X-linked juvenile retinoschisis (XLRS) is the leading cause of macular degeneration in males. This condition is caused by mutations in the *RS1* gene and is, characterized by schisis within the retina. The purpose of this study was to identify the mutations in the *RS1* gene associated with XLRS in an Indian cohort.

**Methods:**

The coding region of *RS1* was analyzed for mutations by polymerase chain reaction-single strand conformational polymorphism (PCR-SSCP) and restriction fragment length polymorphism (RFLP) analysis in six unrelated subjects clinically diagnosed as having XLRS and in their available family members. Direct sequencing was performed for all samples that displayed an electrophoretic mobility shift in SSCP gel.

**Results:**

Mutation analysis of *RS1* gene revealed five mutations in exon 6 like c.574C>T, c.583A>G, c.608C>T, c.617G>A, and c.637C>T, respectively, among them four missense mutations, one nonsense mutation, and two novel sequence variations. These mutations were found in individuals who exhibited clinical features of bilateral foveal and peripheral retinoschisis consistent with XLRS. The mutations were absent in the 100 age matched control samples analyzed.

**Conclusions:**

This is the first report of mutations in *RS1* to be associated with XLRS in the Indian population. The identified genetic variations, phenotype and genotype correlations were consistent with other studies. Identification of the causative mutation in patients with XLRS is helpful in confirming the diagnosis and in counseling of family members.

## Introduction

X-linked juvenile retinoschisis (XLRS) is the most common cause of macular dystrophy in males, resulting in vision loss early in life [1]. Estimations of its prevalence range from 1:5000 to 1:25000 [2]. It is characterized by a mild to severe decrease in visual acuity, foveal schisis due to a splitting of the retinal layers, progressive macular atrophy, and reduction in the ERG b-wave [3]. Lesions in the peripheral retina have been observed in half the cases. Clinically the presentation is variable; most patients present with progressive visual impairment between five and ten years of age, but a proportion of patients present in infancy with squint, bilateral nystagmus and highly elevated bullous retinoschisis (RS) [4,5]. During the course of the disease, complications such as retinal detachment, vitreous hemorrhage and neovascular glaucoma can arise, leading to a poor visual outcome [3,6].

The *RS1* gene responsible for XLRS was identified by positional cloning and found to encode a 24-kDa protein called retinoschisin, or RS1 [7], which is secreted from photoreceptor and bipolar cells, as a disulfide-linked oligomeric complex [8]. The function of retinoschisin is unknown. The *RSI* gene contains a discoidin domain. Disease-causing mutations are clustered in regions that encode this domain, suggesting that it is crucial for the normal function of retinoschisin. Several deleterious gene mutations have been reported in different ethnic groups [1]. The *RS1* gene maps to the distal short arm of the X chromosome (Xp22) [9] and consists of six exons that span approximately 15 kb of genomic DNA. Over 130 different mutations in *RS1* are associated with retinoschisin. These include small intronic deletions, nonsense and missense mutations, frameshift insertions, deletions, and splice site mutations. Most are missense mutations in exons 4-6 encoding the discoidin domain. A large number of these involve cysteine residues. Most mutations in the discoidin domain result in protein misfolding and intracellular retention by the endoplasmic reticulum (ER) quality control system [6]. The discoidin domain is implicated in cell-cell adhesion and phospholipid binding, a function which is in agreement with the observed splitting of the retina in XLRS patients, indicating that *RS1* is important during retinal development [1,8].

Some published reports have described the clinical features with defined mutations in the *RS1* gene [10-12]. In this study, we discuss five mutations, two of which have not been previously described, and we determine the clinical phenotype associated with these genotypes in six patients.

## Methods

### Clinical evaluation

Subjects suspected to have XLRS were recruited to the study from outpatients presenting at a tertiary care eye hospital. Eligible participants were prospectively evaluated by visual acuity tests, indirect ophthalmoscopy, slit lamp biomicroscopy. Ganzfeld electroretinography (ERG), and optical coherence tomography (OCT) with (Stratus OCT), version 4.0.1, (Carl Zeiss Meditec, Dublin, CA). The clinical diagnosis of XLRS was based on the presence of foveal schisis with/without peripheral schisis, supported by typical findings on ERG and OCT, as well as a positive family history. Family members, where available, were screened for fundus findings suggestive of XLRS; further evaluation was similar when clinically suggested. The study protocol, which followed the tenets of the Declaration of Helsinki, was approved by the institutional review board. Informed consent was obtained from the patients and their family members prior to their evaluation in the course of the study. The clinical management was conservative in all cases. Six unrelated male patients with XLRS, their available family members, and 100 healthy control subjects, were recruited for the study.

### Genetic evaluation

DNA extraction and single strand conformation polymorphism (SSCP) was performed as follows: Venous blood (5 ml) was collected for genomic DNA extraction using the salt precipitation method described by Miller et al. [13]. For all probands, polymerase chain reaction (PCR) was carried out to amplify the exonic regions of *RS1*, using the primers in Table 1 [1].

**Table 1:** Sequences of oligonucleotide primers used in the present study on *RS1* gene mutations.

**Exon**	**Primer (5'-3')**	**Product size (bp)**
1	F: CTCAGCCAAAGACCTAAGAAC	216
	R: GTATGCAATGAATGTCAATGG	
2	F: GTGATGCTGTTGGATTTCTC	177
	R: CAAAGTGATAGTCCTCTATG	
3	F: CTGCCCTGCCTCTCTGGTTG	178
	R: GGTGTTCCCAATGACTGTTCC	
4	F: GGTGCTTGTTGAGTATTGAG	219
	R: AAAATCCCCGGGCCCTGC	
5	F: GAGTCTCTCGGTGACTCGGT	263
	R: GAGCTGAAGTTGGTTTGGGA	
6	F: CCGATGTGATGGTGACAGG	262
	R: TGTGTGAGGGGGTCCCCTA	

This table describes, the primers used to amplify the coding regions of the *RS1* gene (Retinoschisis consortium). Below bp indicates base pair.

The amplified products were diluted with an equal volume of loading buffer (95% formamide: Sd Fine chemical laboratories, Pvt. Ltd., Mumbai, India, 10 mM NaOH: Qualigens, Mumbai, India, 0.05% bromophenol blue: Himedia Laboratoris, Pvt. Ltd., Mumbai, India, 0.05% xylene cyanol: LOBA CHEMIE, Mumbai, India) and heated at 98 °C for 5 min followed by snap cooling on ice . Denatured amplicons were loaded onto 12% polyacrylamide gel and electrophoresed at 800 V for 8-10 h at room temperature. The gels were silver stained according to the modified protocol of Bassam et al. [14].

### DNA sequence analysis

PCR products that demonstrated a mobility shift in SSCP gels were re-amplified, using the same set of primers and column purified using Perfect-prep gel clean-up kit (Eppendorf, Hamburg, Germany). Next we performed the sequencing in Balgach, Switzerland at Microsynth using dye terminator chemistry on an Applied Biosystem (ABI) model 3730 automated sequencer (Microsynth, Balgach, Switzerland). RFLP analysis was carried out to identify the c.637C>T mutation using *MSP1* restriction enzyme.

## Results

Six male patients with XLRS and their available family members were enrolled in the study and analyzed for *RS1* mutations, which revealed significant changes. Five genetic variations were identified: c.574C>T, c.583A>G, c.608C>T, c.617G>A, and c.637C>T. Two of these, c.583A>G and c.617G>A, are novel. Similar to previous reports, all the mutations were missense and nonsense, and they were clustered in exon 6 encoding the discoidin motif. The amino acid change of each identified mutation is given in Table 2. We did not find any polymorphic variations in patients as well as in controls. The clinical phenotypes of genetically analyzed patients are summarized in Table 3. The range of age was 10-30 years. OCT showed multilayered schitic cavities in the macula, predominantly in all participants OCT showed multilayered schitic cavities in the macula, predominantly in the plexiform layers. ERG findings showed selective b-wave suppression in all patients. The pathogenic effects of the mutations identified were confirmed by excluding their presence in 100 normal controls, of whom (more than 60% were males).

**Table 2:** Genetic variations in patients with X-linked juvenile retinoschisis.

Patient ID	Nucleotide change	Amino acid change	Exon
1	574C>T	Pro192Ser	6
2	583A>G	Ile195Val*	6
3	608C>T	Pro203Leu	6
4	617G>A	Trp206X*	6
5	637C>T	Arg213Trp	6
6	637C>T	Arg213Trp	6

This table shows the mutations and their corresponding aminoacid changes identified in this study. Asterisk (*) marks a novel genetic variation. The other mutations were previously reported by the Retinoschisis Consortium.

**Table 3:** Clinical characteristics of patients with X-linked retinoschisis.

Patient ID	BCVA	Age (yrs)/ Sex	Foveal schisis	Peripheral schisis	ERG	OCT
1	6/18:6/60	10/M	OD	OU	Negative OU	Schitic cavities OU: full-thickness MH OS
2	6/60: 6/60	11/M	OU	OU	Negative OU	Schitic cavities OU
3	6/18:6/18	20/M	OU		Negative OU	Schitic cavities OU
4	5/60: 6/60	10/M	OU	OU	Both waveforms suppressed (a>b) OD: Negative ERG OS	Schitic cavities OU: detached retina OD
5	6/6: 6/36	17/M	OD	OU	Negative OU	Schitic cavities OU; foveal atrophy OS
6	6/24;6/36	30/M	OU	OU	Negative OU	Schitic cavities OU

The following abbreviations were used: macular hole (MH), vitreous hemorrhage (VH), and rhegmatogenous retinal detachment (RRD). Within table BCVA represents best-corrected visual acuity; ERG represents electroretinography; OCT represents optical coherence tomography.

### Patient 1 with c.574C>T mutation

A c.574C>T mutation was found in a 10-year-old male. His best corrected visual acuity (BCVA) was OD: 6/18, OS: 6/60. He had foveal schisis in his the right eye, and peripheral schisis in both eyes. There was a full-thickness macular hole in his left eye. The clinical findings of this patient have been previously reported elsewhere [15].

### Patient 2 with c.583A>G novel genetic variation

A c.583A>G variation was identified in an 11-year-old male. His BCVA was OD: 6/60, OS: 6/60. Clinical features associated with this mutation were foveal and a temporal peripheral schisis observed bilaterally. He had rhegmatogenous retinal detachment (RRD) in both eyes, although confined to the temporal schisis. To the best of our knowledge, c.583A>G transition leading to the replacement of isoleucine at position 195 by valine has not been previously described and is not highly conserved across the species.

### Patient 3 with c.608C>T mutation

A c.608C>T mutation was observed in a 20-year-old male. His BCVA was OD: 6/18, OS: 6/18. He had bilateral foveal schisis, with normal periphery.

### Patient 4 with c.617G>A novel nonsense variation

**Figure 1:**
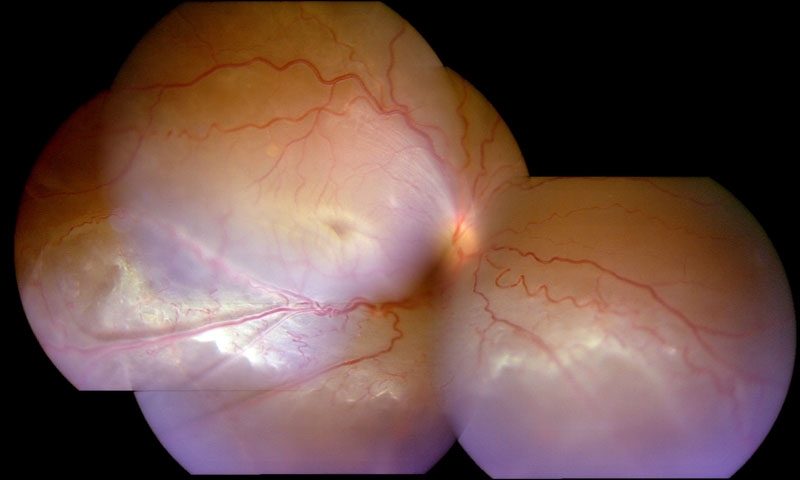
Fundus photography of patient 4 with X-linked juvenile retinoschisis. Composite fundus photograph of the right eye of patient 4 showing total rhegmatogenous retinal detachment secondary to inferotemporal schisis, which is evident as elevated inferotemporal retinal vessels (vitreous veils). The foveal schisis is obscured by the detachment.

A c.617G>A nonsense mutation was found in a 10-year-old male. His BCVA was OD: 5/60, OS: 6/60. He had foveal and peripheral schisis in both eyes. His ERG findings showed suppression of a and b-waves in his right eye, although b-wave amplitude was depressed to a greater extent. He had total RRD in his right eye (Figure 1). Surgical intervention was proposed; but was declined by the patient.

**Figure 2:**
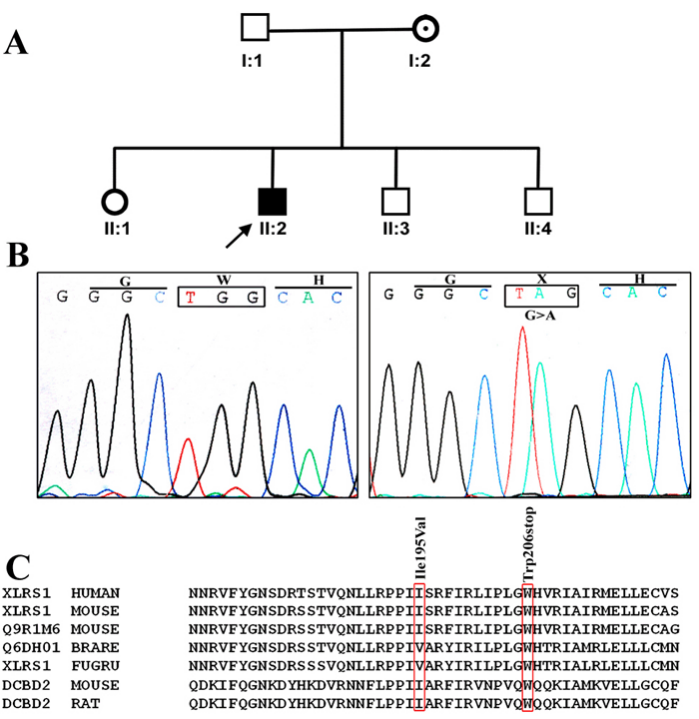
Genetic analysis of patient 4 with X-linked juvenile retinoschisis. **A**: Family pedigree of patient 4, who exhibited a novel c.617G>A mutation. The arrow indicates proband. The shaded box denotes that the proband affected with retinoschisis. The dotted circle indicates carrier. **B**: This sequence chromatogram compares the novel c.617G>A nonsense mutation with the sequence derived from control. The DNA of proband II: 2 revealed a homozygous G to A transition in exon 6 of *RS1* gene that leads to the replacement of aminoacid tryptophan to stop codon at position 206. **C**: A multiple sequence alignment for I195V and W206X novel genetic variants of *RS1* gene, in different species and compared with other retinoschisin and discoidin domain proteins. The amino acids boxed in red indicate the position of I195V, and W206X. The aminoacid tryptophan is highly conserved among the various species.

The parents (I-1, I-2) and brother (II-3) of patient 4 also participated in this study (Figure 2A). His mother (I-2) was heterozygous for the same mutation, but the father (I-1) and brother were unaffected. Mutation analysis of *RS1* and sequencing result showed a c.617G>A transition that resulted in a nonsense mutation (Figure 2B) where an evolutionarily highly conserved tryptophan was replaced by a stop codon X at position 206 (Figure 2C).

### Patient 5 with c.637C>T transition

**Figure 3:**
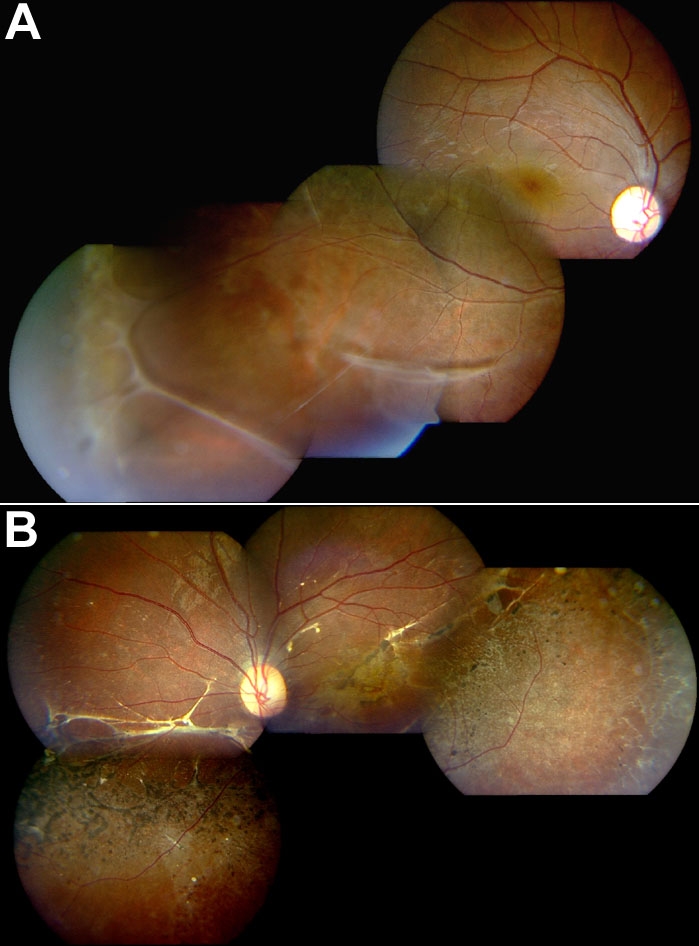
Fundus photography of patient 5 with X-linked juvenile retinoschisis. **A**: Composite fundus photographs of the right eye of patient 5 showing foveal schisis, as well as peripheral vitreous veils. **B**: Composite fundus photograph of the left eye of the same patient, showing spontaneously settled inferotemporal retinal detachment involving macula, with consequent foveal atrophy.

A c.637C>T transition was observed in a 17-year-old male. His BCVA was OD: 6/6, OS: 6/36. He has foveal schisis in his right eye, and peripheral schisis in both eyes (Figure 3A). He demonstrated a spontaneously settled inferotemporal RRD involving the left macula, with consequent foveal atrophy (Figure 3B). His right eye showed evidence of foveal and parafoveal atrophy.

### Patient 6 with c.637C>T transition

A c.637C>T transition mutation was also identified in patient 6, a 30-year-old male. His BCVA was OD: 6/24, OS: 6/36. He had foveal and peripheral schisis in both eyes. He developed a vitreous hemorrhage (due to avulsion of floating vessels) in his left eye, which settled spontaneously over two months.

## Discussion

Mutations of the *RS1* gene are known to cause many cases of inherited and sporadic XLRS. Previous reports have described the clinical features in families with defined mutations in the *RS1* gene [1,10-12]. In the present study, patients with the clinical phenotype of XLRS had five different genetic variations in the *RS1* gene. All patients had typical clinical features, such as foveal schisis (with or without peripheral RS) and ERG b-wave suppression. Four patients had bilateral foveal schisis. Five patients had a temporal peripheral schisis. Three patients had RRD with variable extent. In addition to that, two patients had atypical findings. The right eye of patient 5 demonstrated evidence of foveal and parafoveal atrophy. The left eye of patient 1 exhibited a full-thickness macular hole. Four of the genetic variants identified in this study were noted to be sporadic cases, and one novel genetic variant c.617G>A was identified in a family.

A c.617G>A novel nonsense mutation was identifiedin patient 4. His mother had the same mutation, although in the heterozygous condition, and his brother and father were unaffected. The co-segregation of this gene mutation with the RS phenotype and the RS carrier status as well as its complete absence in normal control chromosomes indicate this genetic change is responsible for the RS pathology in this family and that the disease is transmitted as an X-linked recessive trait. Another non-sense mutation involving a different nucleotide c.618G>A in the same codon was previously reported in a French population [1]. Our results are also consistent with this report. Highly conserved tryptophan was replaced by stop codon X at position 206, causing premature termination that might truncate the protein retinoschisin. ERG findings showed suppression of a and b waves. Total RRD was observed that might be due to loss of function of the protein due to premature termination.

Patient 2 was found to have a c.583A>G novel genetic variation, leading to the replacement of non-conserved amino acid isoleucine at position 195 by valine. This patient had typical retinoschisis clinical findings.

All the identified mutations are present in the hotspot region at exon 6 reported by the retinoschisis Consortium [1]. These belong to the discoidin motif of retinoschisin protein. Retinoschisin is a soluble secretory protein predicted to have a globular conformation [16]. Recent in vitro expression analysis revealed that the pathologic basis of RS is intracellular retention and defective secretion of the mutant retinoschisin as a result of protein misfolding [17]. Many missense and protein-truncating mutations of the causative gene *RS1* have now been identified and are thought to be inactivating. These mutations are mainly located in exons 4-6. An alignment of 32 discoidin domain proteins was constructed to reveal the consensus sequence and to deduce the functional importance of the identified [1].

Our data provides evidence that the five *RS1* gene mutations identified in our series of XLRS patients might lead to disruption of gene expression. Further functional insights needs to be elucidated. The genotypic approach will provide further understanding of the influence of genes in the pathogenesis of clinical disease and the correlation of the genotype with the phenotype. Carriers of mutant genes can be identified, enabling the identification of offspring at risk for specific disease as well as precise diagnosis before the development of typical, clinically recognizable phenotypes.
